# The relationship between dairy products intake and breast cancer incidence: a meta-analysis of observational studies

**DOI:** 10.1186/s12885-021-08854-w

**Published:** 2021-10-15

**Authors:** Yujing He, Qinghua Tao, Feifei Zhou, Yuexiu Si, Rongrong Fu, Binbin Xu, Jiaxuan Xu, Xiangyuan Li, Bangsheng Chen

**Affiliations:** 1 The Second Clinical Medical College, Zhejiang Chinese Medical University, Zhejiang, Hangzhou China; 2 Emergency Medical Center, Ningbo Yinzhou No. 2 Hospital, Ningbo, Zhejiang, China; 3 School of Basic Medical Sciences, Zhejiang Chinese Medical University, Zhejiang, Hangzhou China; 4 The First Clinical Medical College, Zhejiang Chinese Medical University, Zhejiang, Hangzhou China; 5 Department of Nutrition, HwaMei Hospital, University of Chinese Academy of Sciences, Ningbo, Zhejiang, China

**Keywords:** Breast cancer, Dairy products, Risk assessment, Meta-analysis

## Abstract

**Background:**

The effect of dairy products intake on breast cancer (BC) is highly controversial. This study aims to investigate the relationship between dairy intake and BC incidence.

**Methods:**

A search was carried out in PubMed, EBSCO, Web of Science, and Cochrane Library databases before January 2021. The primary objective was the risk of BC and intake of dairy products were exposure variables.

**Results:**

The meta-analysis comprised 36 articles with 1,019,232 participants. Total dairy products have a protective effect on female population (hazard ratio (HR) =0.95, 95% confidence interval (CI) =0.91–0.99, *p* = 0.019), especially for estrogen receptor-positive (ER+) (HR = 0.79, *p* = 0.002) and progesterone receptor-positive (PR+) BC (HR = 0.75, *p* = 0.027). For ER+/PR+ BC, there is a trend of protection, but it has not reached statistical significance (HR = 0.92, *p* = 0.075). Fermented dairy products can reduce BC risk in postmenopausal population (HR = 0.96, 95%CI = 0.93–0.99, *p* = 0.021), but have no protective effect on premenopausal population (HR = 0.98, 95%CI = 0.94–1.03, *p* = 0.52). Non-fermented dairy products have no significant effect on BC occurrence (*p* > 0.05). High-fat dairy products are harmful to women, without statistical difference (HR = 1.06, 95%CI = 1.00–1.13, *p* = 0.066). On the contrary, low-fat dairy products can protect the premenopausal population (HR = 0.94, 95%CI = 0.89–1.00, *p* = 0.048).

**Conclusion:**

The intake of dairy products can overall reduce BC risk in the female population, but different dairy products have varying effects on different BC subtypes and menopausal status.

**Supplementary Information:**

The online version contains supplementary material available at 10.1186/s12885-021-08854-w.

## Introduction

Breast cancer (BC) remains a worldwide public health dilemma [1–4]. Based on the latest GLOBOCAN data, female BC has surpassed lung cancer as cancer with the highest incidence worldwide [5]. Meanwhile, BC is also the most common cause of death in women [6–8]. Both endogenous factors (genetic inheritance [9] and genetic mutations [10]) and exogenous factors (lifestyle [11], environmental factors [12], and reproductive factors [13, 14]) can influence the BC occurrence [2, 3, 15].

Nutrition and lifestyle habits are among the most easily modifiable aspects of people’s lives which are considered effective prevention strategies for cancer [16–19]. On the one hand, lifestyle habits such as maintaining circadian stability [20], reducing sedentary behavior [21], little alcohol consumption [22], and little smoking [23] are considered to reduce BC risk. On the other hand, diet choices have an impact on health and cancer incidence rate [24]. Numerous studies on the relationship between diet and BC have recently been conducted [25, 26]. For instance, plant-based dietary pattern [27, 28], low-fat dietary pattern [29], “Prudent” dietary pattern [30], and Mediterranean diet [31] were inversely associated with BC risk. In addition, several studies have been conducted on the effects of dairy products [32], soy products [33], fruits [34], vegetables [35], dietary fiber [36], and mushrooms [37] on BC risk rates.

Numerous studies regarding the impact of dairy products on BC risk rates remain inconsistent [38–40]. Valeria Pala et al. [41] found inconsistent correlations between BC risk and dairy products, whereas McCullough ML et al. [40] discovered an inverse relationship between daily consumption of two or more servings of dairy products and BC risk. Kato I et al. [42] conducted a study on the impact of dairy products on BC and concluded that dairy products increase BC risk, while Emmanuelle Kesse Guyot et al. [43] found that BC risk was lower in those with high total consumption of dairy. In addition, there is controversy in the findings of studies regarding the impact of various dairy types (fermented dairy products, high-fat dairy products, etc.) on BC risk rates and the effect of the same type of dairy products on risk rates of various BC subtypes (estrogen receptor-positive (ER+), progesterone receptor-positive (PR+), etc.) [44, 45].

Besides that, dairy products contain complex nutritional components, and their consumption is widespread in daily life [46]. Many studies have indicated that dairy products may also impact health problems such as obesity [47], diabetes [48, 49], cancer [50], and coronary heart disease [51, 52], but whether dairy products play a protective or harmful role against BC occurrence in female population is still controversial.

To provide preventive strategies for BC-related dietary choices more scientifically, statistical analysis of the available research data is now urgently required to provide reliable evidence for the association between intake of dairy products and BC risk. Therefore, this study aimed to evaluate the association between intake of dairy products and BC risk through meta-analysis.

## Materials and methods

### Article source and search strategy

A search of relevant studies investigating the relationship between dairy products and BC published before January 2021 was carried out in PubMed, EBSCO, Web of Science, and Cochrane Library databases. The keywords we used for searching were “breast cancer” and “dairy”. The complete retrieval formula used to identify the number of studies were (“breast cancer” OR “breast neoplasms” OR “BC”) AND (“dairy” OR “dairy products” OR “milk”). When referencing duplicated literature, the original article was included if the studies were published as an abstract and original article. In addition, if a single study published several articles, only the most recent or the highest quality article was included. The reference lists of retrieved studies and recent reviews have also been reviewed to examine potential inclusive studies. This meta-analysis was conducted according to the Meta-Analysis of Observational Studies in Epidemiology (MOOSE) guidelines [53]. The population, intervention/exposure, comparison, outcome, and setting (PICOS) criteria were used to describe the research question.

### Eligibility criteria

Specific eligible criteria were formulated as follows: inclusion criteria: (1) all included studies are cohort and case-control studies limited to clinical studies only. (2) The main exposure of study was dairy products, and the outcome was BC risk. (3) All studies included available data about hazard ratio (HR) and corresponding 95% confidence interval (CI).

Exclusion criteria: (1) the study had no reference value or control group. (2) The study was conducted on BC population and used mortality or recovery rate as the outcome. (3) The study was not published in English. (4) The study did not contain a full-text article.

### Data extraction

A jointly agreed data collection form was used to extract all data. In order to ensure the objectivity and accuracy of the entered data, two investigators independently extracted data from each study. A third investigator resolved disagreements. Information was extracted as follows: the author’s name, year of publication, study type, country where the study was done, age, follow-up time, number of participants, number of BC cases identified in the study period, dairy type, and outcomes.

### Objectives, outcomes and dairy exposures

The primary objective was the risk of BC and intake of dairy products were exposure variables. The second objective is to explore the effects of various dairy products, including fermented dairy products, non-fermented dairy products, high-fat dairy products and low-fat dairy products, on the incidence of BC. Fermented dairy products refer to acidic dairy products, such as yogurt, cheese, and cream created by fermenting raw milk with lactic acid bacteria or co-fermenting lactic acid bacteria with yeasts using specific microorganisms. Non-fermented dairy products are characterized as those not fermented by microorganisms, such as milk and butter. High-fat dairy products are defined as those with up to 3–5% fat per 100 g of food. Low-fat dairy products were defined as those with a fat content of 1.0–1.5% per 100 g food and nonfat dairy products with a fat content of less than 0.5% per 100 g food. HRs after adjustment for some potential confounderswere uniformly adopted in this meta-study to facilitate the processing of relevant data from the included articles. Common potential confounders include age, exercise, body mass index (BMI), oral contraceptive use, age of menarche, age of menopause, parity, family history of BC, smoking status and alcohol intake.

### Bias risk and study quality assessment

The quality of each included study was evaluated and scored using the Newcastle-Ottawa Quality Assessment Scale (NOS) checklist, a tool used for quality assessment of non-randomized studies, which is composed of eight items classified into three aspects, including selection, comparability, and outcome. The maximum scores of this checklist were nine, and scores between six and nine were identified to higher study quality.

### Statistical analysis

The Stata software version 12 (StataCorp, College Station, Texas, USA) was used to analyze the data. *P*-values less than 0.05 were considered statistically significant. Heterogeneity across included studies was tested by Q statistic and I^2^ statistic, which is a quantitative measure of inconsistency assessment. Following the Cochrane Manual and study characteristics, I^2^ values between 0 and 30% indicated mild or insignificant heterogeneity, those between 30 and 70% indicated moderate heterogeneity, while those between 70 and 100% indicated high or significant heterogeneity [54].

The CI of HR was set at 95% to examine the relationship between dairy consumption and BC risk. A random-effect model was employed to incorporate data due to the inclusion of different dairy products to increase the credibility of results. When more than ten [55, 56] studies were included, sensitivity analysis and publication bias test were performed to evaluate the stability and reliability of the results. Begg’s rank correlation [57] and Egger’s linear regression tests [58] were used to testing publication bias.

## Results

### Literature search

**Fig. 1:**
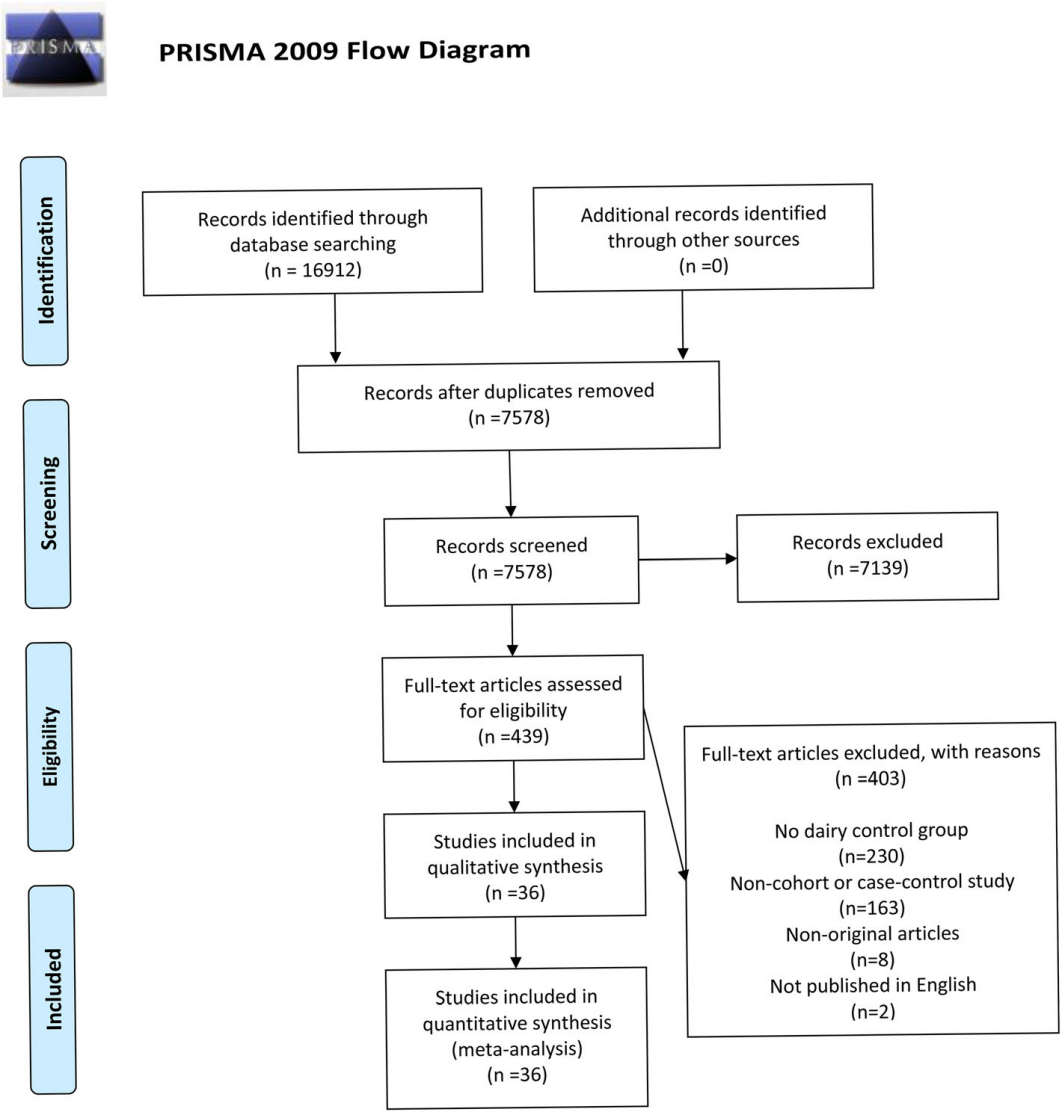
A schematic flow for the selection of articles included in this meta-analysis

A total of 16,912 relevant articles were identified based on retrieval formula described in the methods section by initial search in PubMed, EBSCO, Web of Science, and Cochrane Library database. No additional records were identified through other sources. A total of 9334 duplicated articles were removed, and 7139 articles were excluded according to their titles or abstracts. The remaining 439 articles were scrutinized through full-text reading. Among them, 403 articles were eliminated because of no dairy control group (*n* = 230), non-cohort or case-control study (*n* = 163), non-original articles (*n* = 8), and non-English language (n = 2). Eventually, 36 articles [24, 39–45, 59–86] composed of 1,019,232 participants were selected for this meta-analysis. Figure 1 shows the flow diagram about the selection of articles.

### Study characteristics

All articles were observational studies that record the BC risk in women with dairy products as the exposure factor. Of the 36 included studies, 14 were cohort studies (912,975 participants and 25,097 BC cases), and 22 were case-control studies (106,257 participants and 18,543 BC cases). Among them, 13 studies were conducted in Europe, 11 in North America (9 studies were conducted in the United States), 11 in Asia, and 2 in South America. All studies were published between 1986 and 2020, with follow-up periods ranging from 1.5 to 22.2 years. Most studies do not make special requirements for included participants, two studies were only conducted on nurses, and one study was only conducted in premenopausal population. Regarding age at recruitment, five studies did not set an upper age limit, and two studies did not set a lower age limit. In addition, the adjustment of potential confounding factors varied in different studies. Common adjustment parameters in the included studies included age, BMI, family history of BC, hormone replacement therapy (HRT), reproductive factors, total energy intake, smoking, and alcohol drinking. In order to collect data and evaluate relevant exposure factors, 21 studies chose a food frequency questionnaire (FFQ), 11 studies selected a diet questionnaire, and 3 studies chose a home visit or in-depth interview. Table 1 and Supplementary Table 1 contain detailed characteristics of the included studies.

**Table 1:** Characteristics of included clinical trials in the meta-analysis

Author, year	Country	Age of recruitment (year)	Age of analysis (year)	Follow-up time (year)	No. of cases	No. of participants	Characteristics
Kaluza, 2020	Swedish	48–83	63.6 ± 9.4	16.6	1870	33,780	The Swedish Mammography Cohort
Farvid, 2018	Amercia	25–42	37.1 ± 4.6	NA	4509	90,503	The Nurses’ Health Study II cohort
Fraser, 2020	Amercia	≥30	57.1 ± 3.5	7.9	1057	25,795	The Adventist Health Study-2
Genkinger, 2013	Amercia	21–69	NA	12	1268	59,027	The Black Women’s Health Study
Kesse-Guyot, 2007	French	35–60	51.2 ± 6.3	8	92	3627	The French SU.VI.MAX Study
Knekt, 1996	Finland	15–90	39.1 ± 16.5	25	88	4697	The Finnish Mobile Clinic Health Examination Survey
Shin, 2002	Amercia	35–70	46.7 ± 7.6	16.5	3482	88,691	The Nurses’Health Study
Pala, 2009	European	25–70	50.8 ± 8.4	8.8	7119	319,826	Cancer and Nutrition cohort
Marcondes, 2019	Netherlands	≥55	68.3 ± 8.7	17	199	3092	The Rotterdam Study
Shin, 2019	Korean	40–69	52.7 ± 7.9	6.3	359	78,320	The Health Examinees-Gem
Hjartåker, 2010	Norway	26–64	56.8 ± 6.7	8.6	1407	64,904	The Norwegian Women and Cancer study
McCullough, 2005	Amercia	50–74	62.2 ± 4.2	9	2855	97,786	The Cancer Prevention Study II Nutrition Cohort
Gaard, 1995	England	35–49	43.5 ± 8.4	7–13	248	25,892	The Norwegian National Health Screening Services
Wirfält, 2011	Sweden	45–73	NA	10.3	544	17,035	The Malmo Diet and Cancer Cohort
Yu, 2019	China	25–70	NA	NA	1286	2747	NA
Franceschi, 1995	Italy	23–74	55.3 ± 4.3	NA	2569	55,175	NA
Galván-Salazar, 2015	Mexican	40–65	50.7 ± 7.5	NA	97	201	NA
Potischman, 2002	Amercia	20–44	NA	NA	568	2019	NA
Jayalekshmi, 2009	India	≥20	46.4 ± 3.6	NA	792	1056	NA
Hirose, 2003	Japan	≥30	NA	NA	2385	21,398	NA
Bao, 2012	China	25–70	NA	NA	3443	6917	The Shanghai Breast Cancer Study
Van ‘t Veer, 1991	Netherlands	25–44, 55–64	NA	1.5	133	422	NA
Bahadoran, 2013	Iran	30–65	46.2 ± 8.9	NA	100	275	NA
Toniolo, 1994	Amercia	35–65	52.2 ± 8.4	22.2	180	1009	The New York University Women’s Health Study
Plagens-Rotman, 2017	Poland	21–84	52.5 ± 11.1	NA	79	762	NA
Lima, 2008	Brazil	30–80	56.3 ± 6.8	NA	89	183	NA
Lê, 1986	French	40–70	NA	NA	1010	2960	NA
Shannon, 2003	Amercia	50–64	NA	NA	440	810	NA
Kato, 1992	Japan	≥20	NA	NA	908	1816	NA
Mobarakeh, 2014	Iran	20–65	40.2 ± 10.1	NA	53	93	NA
Potischman, 1998	Amercia	≤45	NA	NA	1647	3148	NA
McCann, 2017	Amercia	35–75	45.5 ± 5.9	NA	1857	3059	NA
Zhang, 2011	China	25–70	47.0 ± 9.5	NA	438	876	NA
van’t Veer, 1989	Netherlands	25–64	NA	NA	133	548	NA
Ronco, 2002	Uruguay	≤85	NA	NA	111	333	NA
Ahmadnia, 2016	Iran	36–50	NA	NA	225	450	NA

NA: not available

### Analysis about the effect of total dairy products on BC

Twenty-five (726,673 participants) studies recorded data about BC risk of total dairy products on female population, with 30,334 participants newly diagnosed with BC over the follow-up time. The analysis indicated that those who consumed dairy products had a significantly lower BC risk than those who never or rarely consumed dairy products (HR = 0.95, 95% CI = 0.91–0.99, *p* = 0.019) with moderate heterogeneity (I^2^ = 62.3%), indicating that dairy product intake was protective for female population and can reduce BC risk. However, the consumption of dairy products has no effect on premenopausal (HR = 1.02, 95% CI = 0.97–1.09, *p* = 0.37) (I^2^ = 5.3%) and postmenopausal women (HR = 1.00, 95% CI = 0.97–1.04, *p* = 0.83) (I^2^ = 0%). Four studies (185,355 participants) were grouped by ER status, three studies (87,569 participants) were grouped by PR status, and five studies (184,989 participants) were grouped by hormone receptor status (included ER and PR). The meta-analysis found that consuming dairy products significantly reduces the risk of hormone-receptor-positive BC, either ER+ BC (HR = 0.79, *p* = 0.002) or PR+ BC (HR = 0.75, *p* = 0.027). For ER+/PR+ BC, a trend was observed towards lower risk with consuming dairy products, but the difference was not statistically significant (HR = 0.92, *p* = 0.075). In addition, the consumption of dairy products had no effect on the incidence of ER- BC (HR = 1.06, *p* = 0.42), PR- BC (HR = 1.05, *p* = 0.56) and ER−/PR- BC (HR = 0.96, *p* = 0.36) BC. Table 2 contains the detailed data.

**Table 2:** Effects of dairy products on breast cancer incidence

Subgroup analysis	No. of studies	Cases	Participants	HR	95%CI	*p*	Heterogeneity (I^2^) (%)
All populations	25	30,334	726,673	0.95	0.91–0.99	*0.019*	63.2
ER+ breast cancer	4	6466	185,355	0.79	0.68–0.92	*0.002*	66.4
ER- breast cancer	4	6466	185,355	1.06	0.92–1.22	0.42	0
PR+ breast cancer	3	3611	87,569	0.75	0.58–0.97	*0.027*	73.9
PR- breast cancer	3	3611	87,569	1.05	0.90–1.21	0.56	0
ER+/PR+ breast cancer	5	11,563	184,989	0.92	0.85–1.01	0.075	73.0
ER−/PR- breast cancer	5	11,563	184,989	0.96	0.88–1.05	0.36	32.5
Premenopausal population	8	14,863	653,249	1.02	0.97–1.07	0.37	5.9
Postmenopausal population	8	14,863	653,249	1.00	0.97–1.04	0.83	0
Fermented dairy products	14	16,280	496,747	0.98	0.95–1.02	0.25	41.9
Fermented dairy products for ER+/PR+ breast cancer	2	2414	50,815	1.01	0.97–1.05	0.62	12.3
Fermented dairy products for ER−/PR- breast cancer	2	2414	50,815	1.03	0.92–1.15	0.63	37.4
Fermented dairy products for the premenopausal population	6	14,425	561,870	0.98	0.94–1.03	0.52	0
Fermented dairy products for the postmenopausal population	6	14,425	561,870	0.96	0.93–0.99	*0.021*	0
Non-fermented dairy products	13	17,145	594,111	0.99	0.94–1.03	0.54	0
Non-fermented dairy products for the premenopausal population	6	14,425	561,870	1.03	0.97–1.09	0.33	0
Non-fermented dairy products for the postmenopausal population	6	14,425	561,870	1.03	0.98–1.08	0.30	20.2
High fat dairy products	12	19,208	624,176	1.06	1.00–1.13	0.066	63.7
High fat dairy products for premenopausal populations	6	17,873	584,718	1.01	0.96–1.07	0.61	0
High fat dairy products for the postmenopausal population	6	17,873	584,718	1.00	0.95–1.04	0.87	0
Low fat dairy products	11	19,699	615,226	0.98	0.94–1.03	0.50	63.1
Low fat dairy products for the premenopausal population	6	17,873	584,718	0.94	0.89–1.00	*0.048*	33.5
Low fat dairy products for the postmenopausal population	6	17,873	584,718	1.04	0.99–1.09	0.15	22.6

HR: hazard ratio; CI: confidence interval; ER: estrogen receptor; PR: progesterone receptor

### Analysis about the effects of fermented dairy products on BC

In terms of fermented dairy products, 14 studies (496,747 participants) were conducted on female populations, six of which (50,815 participants) were grouped by menopause status, and two of which (561,870 participants) were grouped by hormone receptor status (ER and PR). During the follow-up period, 16,280 participants were newly diagnosed with BC. The analysis demonstrated that fermented dairy products were not statistically significantly protective for women (HR = 0.98, 95%CI = 0.95–1.02), nor for ER+/PR+ BC (HR = 1.01, 95%CI = 0.97–1.05) or ER−/PR- BC (HR = 1.03, 95%CI = 0.92–1.15). A subgroup analysis for menopause status found that although fermented dairy products had no significant effect on the premenopausal population (HR = 0.98, 95%CI = 0.94–1.03, *p* = 0.52), they had a protective effect on the postmenopausal population (HR = 0.96, 95%CI = 0.93–0.99, *p* = 0.021) and could reduce BC risk in the postmenopausal population without heterogeneity (I^2^ = 0%). Table 2 lists the detailed data.

### Analysis about the effects of non-fermented dairy products on BC

Thirteen studies (594,111 participants) explored the effect of non-fermented dairy products on BC risk in female populations, six of which included detailed data on different menopausal conditions. A total of 17,145 participants were newly diagnosed with BC during the study period. The analysis showed that non-fermented dairy products did not affect all populations (HR = 0.99, *p* = 0.54), including premenopausal (HR = 1.03, *p* = 0.33) and postmenopausal population (HR = 1.03, *p* = 0.30) with low heterogeneity. Table 2 includes the detailed data.

### Analysis about the effect of high-fat dairy products on BC

In total, 12 studies investigated the relationship between high-fat dairy products and BC. Six studies compared the risk rates between premenopausal and postmenopausal populations. Among 424,176 participants, 19,208 were newly diagnosed with BC. After meta-analysis, no statistically significant difference was observed in the effect of high-fat dairy products on all populations (HR = 1.06, 95%CI = 1.00–1.13, *p* = 0.066). However, there was a harm trend, indicating that high-fat dairy product consumption tends to increase BC risk in female populations, with moderate heterogeneity (I^2^ = 63.7%). Apart from that, the analysis also revealed that high-fat dairy products did not affect premenopausal (HR = 1.01, *p* = 0.61) and postmenopausal populations (HR = 1.00, *p* = 0.87). The heterogeneity between different studies was all 0%. Table 2 comprises the detailed data.

### Analysis about the effect of low-fat dairy products on BC

Eleven studies (615,226 participants) explored the impact of low-fat dairy products on BC, including six studies (584,718 participants) grouped by menopause. During the study period, During the study period, 19,699 newly detected BC cases were found. According to the meta-analysis, low-fat dairy products were found to have no noticeable effect on all populations (HR = 0.98, 95%CI = 0.94–1.03, *p* = 0.50) and postmenopausal population (HR = 1.04, 95%CI = 0.99–1.09, *p* = 0.15). However, for the premenopausal population, the consumption of low-fat dairy products conferred a statistically significant difference in protection against BC (HR = 0.94, 95%CI = 0.89–1.00, *p* = 0.048) with moderate heterogeneity among studies (I^2^ = 22.6%). Table 2 provides the detailed data.

### Risk of bias assessment

The NOS checklist was adopted to objectively evaluate the quality of included observational studies in this meta-study. According to the quality evaluation results of the investigators, 13 studies out of 36 were rated 9 points, 16 studies were rated 8 points, 5 studies were rated 7 points and 2 studies were rated 6 points. All included studies were of higher quality based on methodology. Supplementary Table 2 explicitly recorded the assessment of risk of bias.

### Analysis of publication bias and sensitivity

Begg’s rank correlation and Egger’s linear regression test were employed to estimate publication bias. Begg’s rank correlation test and Egger’s linear regression test results indicated the absence of publication bias among included articles (*p* > 0.05). The sensitivity analysis suggested that the overall risk assessment was not substantially modified by any single study, revealing the stability of the above results.

## Discussion

Whether dairy products play a protective or harmful role against BC occurrence in female population is still controversial. Review of previous studies, Zang J et al. [87] also conducted a meta-analysis of the relationship between BC and dairy products, but their study did not conduct multifactorial subgroup analysis. In addition, their study considered cheese and butter as the same exposure factor and yogurt as another exposure factor. However, since cheese and yogurt are fermented dairy products and butter is not, they did not analyze fermented dairy products as a whole and misclassified cheese and butter, implying that the result could lack reliability. There was also inadequacy in the meta-analysis of Dong JY et al. [88] First, their studies were only retrieved from PubMed one database, probably ignoring the potentially included studies. Second, they only performed a subgroup analysis on the role of milk and could not provide a comprehensive conclusion.

Compared with previous studies, our study has its own strengths. First, this study included many observational studies with more than one million participants in Asia, Europe, and the Americas. The larger observational population increases the reliability and authenticity of this study’s conclusions. Secondly, this study selected HR data that adjusted the largest number of potential confounding factors for statistical analysis to improve accuracy. Third, the study grouped the abstracted data (by BC type, menopausal status, or type of dairy products) and did subgroup analyses to comprehensively screen the possibility of effects of different dairy products on different populations and different BC types.

According to data analysis, we found that total dairy products have a protective effect on female populations, which can reduce the incidence rate of BC, especially ER+ and PR+ BC. For ER+/PR+ BC, there is a trend of protection without reaching a statistical significance. Fermented dairy products can reduce BC risk in postmenopausal population but have no protective effect on premenopausal population. Non-fermented dairy products have no significant effect on BC occurrence. There is a trend towards harm in the female population with high-fat dairy products. Although a statistically significant difference was not reached, it is suggested that excess high-fat dairy intake may elevate BC incidence. In contrast, low-fat dairy products can protect the premenopausal population and reduce the incidence rate of BC.

Because of their complex composition, no clear mechanism of action has been proposed to explain the contribution of dairy products to BC incidence [17]. Recent works have shown that dairy products have both pro- and anti-carcinogenic effects. Dairy products are rich in calcium, vitamin D, and conjugated linoleic acid (CLA), which affect cell proliferation and differentiation and can inhibit tumor development [89–92]. Conversely, large amounts of fats, saturated fats, and potentially carcinogenic contaminants (pesticides, estrogen metabolites, and growth factors including insulin-like growth factors-1 (IGF-1)) in dairy products increase BC risk [89, 93, 94].

Dairy calcium can have antiproliferative properties by reducing the ability of circulating lipids to induce cell proliferation [92, 95, 96]. Vitamin D was shown to interrupt insulin and IGF-1 and reduce carcinogenesis [97, 98]. Insulin stimulates the elevation of free IGF-1, which can promote cell cycle progression and angiogenesis and has anti-apoptotic properties [43, 79, 99]. CLA is a mixture of positional and geometric isomers of linoleic acid, which are mainly from dairy products (60%) and beef products (32%) [100]. It has anticancer effect in animal experiments and is an effective anticancer drug with cytotoxic effects on BC cells [82, 101], potentially by inhibiting the cyclooxygenase-2 or lipoxygenase pathways or by inducing the expression of apoptotic genes [80, 102]. Accordingly, we speculate that dairy products may have a protective effect on female populations through many of the above pathways to reduce the risk rate of BC. In addition to these, studies have shown that dairy partial ingredients and their bioactive components can exert inhibitory effects on BC by downregulating ER-αexpression and activity, inhibiting proliferation, metastasis, and angiogenesis of breast tumor cells, inducing apoptosis and cell cycle arrest and sensitizing breast tumor cells to radiotherapy and chemotherapy, which may explain the reason why dairy products have apparent protective effects on hormone receptor-positive BC [33, 103–105].

Fermented dairy products, such as yogurt and cheese, have a superior nutritional value over non-fermented milk due to their high concentrations in beneficial bacteria, calcium, riboflavin, vitamin B6, and vitamin B12 [106]. Studies have shown that the probiotic *Lactobacillus acidophilus*, present in yogurt, modulates immune responses against BC in mouse models [107]. By increasing age, the gut exhibits declining levels of bifidobacteria with antitumor effects, which allow toxin-induced tumor growth producing bacteria to thrive, such that postmenopausal populations have lower levels of intestinal probiotics than premenopausal populations [108]. Whereas fermented dairy products can provide probiotics to supplement and balance the gut microbial community, thereby reducing cancer risk [109]. In addition, IGF-I amount that may increase BC risk is significantly reduced in processed heat-treated or fermented dairy products [87, 110]. Combined with the above mechanisms, it may help explain that consumption of fermented dairy products can reduce BC incidence in postmenopausal population. Similarly, non-fermented dairy products that lack probiotics cannot balance the intestinal microbial community and may not affect BC occurrence.

Two potential reasons exist for the detrimental effect of high-fat dairy products on female BC. The first explanation is that high-fat dairy products directly induce BC [111]. Animal experiments have found that a high-fat diet increases mammary epithelial cell proliferation, particularly “hormonally driven” hyperproliferation during mammary gland outgrowth and development in young animals, enhancing the promotion of chemically induced mammary carcinogenesis and increasing BC risk in adulthood [96, 112]. The second explanation is that consuming high-fat dairy products leads to obesity and type 2 diabetes mellitus (T2D), thus increasing the risk of cancer. In particular, BC is more strongly associated with obesity than other cancers [113, 114]. Obese people also have metabolic syndrome, which increases their risk of T2D. Obesity and T2D result in hyperinsulinemia [115, 116], increased insulin-like growth factor [117, 118], hyperglycemia [119], dyslipidemia [120, 121], increased adipokines [122], increased inflammatory cytokines [123] and changes in intestinal microorganisms [124, 125], which may increase the risk of cancer through various mechanisms.

Although consuming fats increases BC risk [126, 127], the effect of different types of fatty acids on BC varies, with studies suggesting that saturated fatty acids are associated with increased BC risk. However, no clear association between total, monounsaturated, or polyunsaturated fatty acids and BC has been observed [128, 129]. Whereas low-fat dairy products differ from high-fat dairy products in the amount of fatty acids obtained by refining full-fat dairy products to extract most saturated fatty acids while preserving unsaturated fatty acids [87]. In addition to this, studies have demonstrated that the association between dairy intake and BC risk is stronger in premenopausal women than in postmenopausal women [43, 79, 130], possibly caused by more robust interactions between calcium, vitamin D, and IGF-1 than in postmenopause [130–132]. This may explain why low-fat dairy products appear to have a risk-lowering impact in the premenopausal population but have no apparent effect in the postmenopausal population.

Although this meta-analysis reached comprehensive and objective conclusions, there are still potential limitations that need to be considered. First, all risk estimates of included studies used multivariate models, but potential risk factors were not adjusted in the same way between studies. Therefore, we cannot exclude the possibility that inadequate control of potential risk confounders contributed to biased results. Second, most studies assessed diet using self-reported FFQs based on their plausibility and reproducibility, although a few used interviews, implying that dietary assessment inevitably produces assessment or measurement error, most likely biasing the findings. Third, consideration should be given to classification biases in dairy products. Because dietary assessments across studies were conducted based on different databases and different FFQs, there may be inconsistencies in the conclusions resulting from different methods of classifying dairy products. Fourth, there may be significant heterogeneity among the included studies due to reported population variations in terms of lifestyle preferences, living locations, and so on. Fifth, not all trials had accessible relevant subgroup data, such as subgroup data by BC type, subgroup data by menopausal status, and thus large-scale observational studies are still required to further validate the relevant conclusions.

Overall, this meta-analysis leads to meaningful conclusions that may allow new recommendations on BC prevention in female populations. Based on the results of this study, we recommend that women should be well suited to consume some dairy products, especially recommending low-fat dairy products for premenopausal women and fermented dairy products for postmenopausal women, minimizing the intake of high-fat dairy products, which may help to reduce the risk of BC.

## Conclusion

This meta-analysis found that total dairy products could reduce BC risk in female populations, especially ER+ BC and PR+ BC. There was a trend toward protection for ER+/PR+ BC that was not statistically significant. In addition, fermented dairy products can reduce BC risk in postmenopausal population, and low-fat dairy products can reduce BC risk in premenopausal population. However, high-fat dairy products are harmful to female population, without statistically significant difference. Based on considerations of relevant limitations, large-scale prospective cohort studies are still required to further confirm the study conclusions.

## Supplementary Information

**Additional file 1 Supplementary Table 1** Characteristics of included clinical trials in the meta-analysis.

**Additional file 2 Supplementary Table 2** Quality assessment of studies included.

## Data Availability

Data supporting findings reported in this study are available in the supplementary materials.

## Abbreviations

BC: breast cancer
ER: estrogen receptor
PR: progesterone receptor
MOOSE: the meta-analysis of observational studies in epidemiology
PICOS: the population, intervention, comparison, outcome and setting criteria
HR: hazard ratio
CI: confidence interval
NOS: the Newcastle-Ottawa quality assessment scale checklist
BMI: body mass index
HRT: hormone replacement therapy
FFQ: food frequency questionnaire
CLA: conjugated linoleic acid
IGF-1: insulin-like growth factors-1
T2D: type 2 diabetes mellitus.
