# Supplementary material for: The relationship between dairy products intake and breast cancer incidence: a meta-analysis of observational studies
Source: BMC Cancer. 2021 Oct 15;21:1109. doi: 10.1186/s12885-021-08854-w (PMC8520314; doi:10.1186/s12885-021-08854-w)
Supplement: Supplementary file 1 — Additional file 1 Supplementary Table 1 Characteristics of included clinical trials in the meta-analysis. [file 12885_2021_8854_MOESM1_ESM.docx]

**Supplementary Table 1**. Characteristics of included clinical trials in the meta-analysis

| Author, year | Study type | Time to recruit patients | Exposure assessment | Dairy type | Adjusting parameters | Reference value (control group) |
| --- | --- | --- | --- | --- | --- | --- |
| Kaluza, 2020 | cohort study | 1997-2016 | FFQ | sour milk/yogurt, hard cheese, cottage cheese, milk | age, education, exercise, height, BMI, oral contraceptive use, HRT use, age of menarche, age of menopause, parity/age at first birth, hypertension, history of benign breast disease, family history of breast cancer, dietary supplement use, intake of alcohol, energy intake | Fermented dairy products:  < 1.0 servings/day  Non-fermented dairy products:  0 servings/day |
| Farvid, 2018 | cohort study | 1998-2013 | FFQ | low-fat milk, high-fat milk, total milk, cheese, ice-cream, yogurt | smoking, race, parity and age at first birth, height, BMI at age 18 years, weight change since age 18, age at menarche, family history of breast cancer, history of benign breast disease, oralcontraceptive use, adolescent alcohol intake, adult alcohol intake, physical activity, adolescent energy intake, hormone use, age at menopause, hormone use andmenopausal status, age at menopause | Total dairy products:  Adolesent group: 1.1 serving/day  Early adulthood: 0.7 serving/day  High fat dairy products:  Adolescent group: 0.6 serving/day  Early adult: 0.2 serving/day  Low fat dairy products:  Adolescent group: 0 serving/day  Early adult: 0.1 serving/day |
| Fraser, 2020 | cohort study | 2002-2007 | FFQ | dairy milk, dairy cheese, yogurt, full-fat dairy milk,  reduced-fat dairy milk | race, family history of breast cancer, time since mammography, menopausal status, oral contraceptive use, nulliparous, smoking, alcohol duration of use, min/week of vigorous physical activity, age at menarche, age at menopause among post-menopausal women, cumulative months of breastfeeding among parous women, number of children among parous women, age at first childbirth among parous women, duration of estrogen replacement therapy, BMI, BMI*menopausal status, total calcium, unprocessed and processed red meats, poultry, fish, seeds, nuts | Total dairy products: 0 kcal/day  Yogurt: 0 kcal/day  Cheese: 0 kcal/day  Milk: never drink  Whole fat dairy products: >0 kcal/day  Fat reducing dairy products: 0 kcal/day |
| Genkinger, 2013 | cohort study | 1995-2007 | FFQ | total milk, whole milk, low-fat milk, hard cheese, yogurt, ice cream | energy intake, age at menarche, BMI, family history of breast cancer, education, parity and age at first live birth, oral contraceptive use, menopausal status, age at menopause, menopausal hormone use, vigorous physical activity, smoking status, alcohol intake | Total dairy products: 0 g/week  Yogurt: 0g/week  Cheese: 0g/week  Cream: 0g/week  Milk: 0 g/week  Whole milk: 0 g/week  Skimmed milk: 0 g/week |
| Kesse-Guyot, 2007 | cohort study | 1994-1995 | 24-hour record every 2  months | total dairy products, milk, yogurt, cheese | educational level, parity, group of treatment, smoking status, overall physical activity, marital status, energy from fat, energy from other sources, alcohol intake, BMI, family history of breast cancer in first degree, menopausal status, dietary energy-adjusted calcium intake | Total dairy products: <165 g  Yogurt: <25g  Cheese: <15g  Cheese: 0g  Milk: <25g |
| Knekt, 1996 | cohort study | 1967-1972 | questionnaire | total dairy products, fermented milk, cream, cheese, milk, butter | age, energy intake,smoking, BMI, number of childbirths, occupation and geographic area, carbohydrates intake, protein intake, fat intake, vitamins and trace elements intake, milk intake | Total dairy products: 486 g/day  Fermented milk: 486 g/day  Cream: 486 g/day  Cheese: 486 g/day  Milk: 486 g/day  Butter: 486 g/day |
| Shin, 2002 | cohort study | 1980-1996 | FFQ | skim/low-fat  milk, sherbet, yogurt, cottage cheese, whole  milk, cream, sour cream, ice cream, cream cheese, other cheese, butter | age, time period, physical activity, history of benign breast disease, family history of breast cancer, height, weight change since age 18, BMI at age 18, age at menarche, parity, age at first birth, alcohol intake, total energy intake, total fat intake, glycemic index, β-carotene intake total active vitamin E intake total calcium intake, total vitamin D intake | Total dairy products: <1 serving/day  Yogurt: 0 serving/day  Cheese: <1 serving/day  Milk: 0 serving/day  High fat dairy products: <4 times/week  Low fat dairy products: <3 times/month |
| Pala, 2009 | cohort study | 1992-2003 | country-specifific food questionnaires | whole milk, semi-skim  milk, skim milk, cheese, butter, milk | energy, height, weight, years of schooling, smoking menopause | Total dairy products: 0 g/day  Cheese: 5.7 g/day  Milk: 0 g/day  Whole milk: 0 g/day  Half fat milk: 0 g/day  Skim milk: 0 g/day |
| Marcondes, 2019 | cohort study | 1989-1993 | semi-quantitative FFQ | milk, yogurt, cheese | energy intake, red meat intake, poultry intake, fatty, lean fish intake, egg, dairy product intake, divided into milk, yogurt and cheese intake, total fat intake, total Fe intake, potential meat or dairy products replacements | Total dairy products: 319.35 g/day  Yogurt: 6.59 g/day  Cheese: 25.06 g/day  Milk: 35.6 g/day |
| Shin, 2019 | cohort study | 2004-2013 | FFQ | milk (any type) | BMI, total energy intake, educational level, parity, age at first birth, age at menarche, oral contraceptive use, regular exercise, alcohol consumption, the presence of a family history of breast cancer | Total dairy products: <1 serving/week |
| Hjartåker, 2010 | cohort study | 1996-2006 | semi-quantitative FFQ | low-fat milk, skimmed milk, yogurt, white cheese | age, energy intake, alcohol intake, height, weight increase since age 18, level of physical activity, years of education, maternal history of breast cancer, mammography practice, age at menarche, number of children and age at first birth, use of oral contraceptives | Total dairy products: <107.2 g/day  Yogurt: 0 g/day  Cheese: <6.0 g/day  Milk: <49.1 g day |
| McCullough, 2005 | cohort study | 1992/1993-2001 | semi-quantitative FFQ | whole milk, low fat milk, skim milk, low fat cheese, yogurt, low fat yogurt, ice cream | age, energy, history of breast cyst, family history of breast cancer, height, weight gain since age 18, alcohol use, race, age at menopause, age at first birth and number of live births, education, mammography history, HRT | Total dairy products: <0.5 servings/d  Milk: 0 servings/day  High fat dairy products: <0.3 servings/week  Low fat dairy products: <0.5 servings/day |
| Gaard, 1995 | cohort study | 1977-1983 | semi-quantitative FFQ | milk, whole milk | age, attained age, height, BMI, menopausal status, smoking, energy intake | Whole milk: 1 glasses/day |
| Wirfält, 2011 | cohort study | 1991-1996 | 7-day menu book, diet questionnaire and 45-min diet history interview | butter-based margarine, vegetable oil margarine, lowfat margarine, yogurt, regular milk, lowfat milk, cream, ice-cream, cheese, cottage cheese | heduration of oral contraceptive use, age at menarche, age at menopause, parity, age at birth of first child, height, weight, BMI, waist circumference, education, alcohol habits, house-hold activity, leisure time physical activity, work activity | Butter based margarine: 0 g  Vegetable oil margarine: 2.8 g  Low fat margarine: 0 g  Yogurt: 0 g  Cream: 0 g  Cheese: 11.4 g  Cheese: 0 g  Milk: 3.6 g  Low fat milk: 0 g |
| Yu, 2019 | case-control study | 2012-2013 | FFQ | milk (any type), cheese, butter, yogurt | residence, age, education, family monthly income, BMI, age of menarche, number of births, age at first childbirth, breast feeding, menopausal status, family history of breast cancer in first degree relatives , cigarette smoking, alcohol drinking, physical activity | Total dairy products: <1 days/week |
| Franceschi, 1995 | case-control study | 1991-1994 | FFQ | milk, cheese | age, centre, education, parity, energy, alcohol intake | Cheese: 2.3 servings/week  Milk: 0.8 servings/week |
| Galván-Salazar, 2015 | case-control study | 2011-2012 | questionnaire | milk (any type) | Gestation, lactation | Total dairy products: 0 g |
| Potischman, 2002 | case-control study | 1991-1992 | FFQ | margarine, butter, milk, ice-cream | age, study site, race, education, alcohol consumption, years of oral contraceptive use, smoking status, BMI, energy | Total dairy products: <7.0 times/week |
| Jayalekshmi, 2009 | case-control study | 1990-2004 | questionnaire | milk (any type) | education levels, marital status, income, occupation, age at marriage, age at first and last pregnancy, number of pregnancies, age at puberty and type of menopause, vegetarian use, non-vegetarian use, and consumption of roots and tubers, tapioca, chicken meat, milk | Total dairy products: occasional |
| Hirose, 2003 | case-control study | 1988-2000 | questionnaire | milk (any type) | occupation, medical history, height, weight, weight at around 20 years of age, marital status, family history, smoking and drinking habits, dietary habits, sleeping habits, physical exercise, reproductive history | Total dairy products: almost never |
| Bao, 2012 | case-control study | 1996-2005 | FFQ | milk (any type) | total energy intake, age, education level, ever diagnosed with benign breast disease, first-degree family history of breast cancer, participation in regular exercise, BMI, study phase, age at menarche, menopausal status, parity, total meat intake, total fruit intake, total vegetable intake | Total dairy products: <7.39 g/day |
| Van 't Veer, 1991 | case-control study | 1985-1987 | 2-hour home visit | yogurt, cheese, sour cream | age, dietary fat intake, alcohol intake, history of benign breast disease, first- and second-degree familial history, smoking habits, educational level of the women, use of oral contraceptives, age at menarche, age at first full-term pregnancy, parity, BMI, geographical area | Fermented dairy products: <464g |
| Bahadoran, 2013 | case-control study | 2010 | semi-quantitative FFQ | yogurt, yogurt  drink, cheese, milk, cream, kashk, whole milk, low-fat milk | age, BMI, educational level, occupation, use of alcohol and tobacco, age at menarche, marital status, age at fifirst pregnancy, number of full-term pregnancies, menopause status, family history of breast cancer, use of oral contraceptive pills (OCP), use of bra, life satisfaction, physical activity, energy intake, energy density of diet | Total dairy products: 183±19 g/day  Fermented dairy products: 137±24 g/day  Non-fermented dairy products: 61±16 g/day  High fat dairy products: 58±14 g/day  Low fat dairy products: 26±22 g/day |
| Toniolo, 1994 | case-control study | 1985-1991 | questionnaire | milk (any type) | height, Quetelet index, age at menarche, age at first full-term pregnancy, number of full-term pregnancies, first-degree family history of breast cancer, history of benign breast conditions, race, religion | Total dairy products: 42 g/day |
| Plagens-Rotman, 2017 | case-control study | 2011-2013 | questionnaire | milk, condensed milk, yogurt, cheese, cream | demographic characteristics, alcohol consumption, smoking | Total dairy products: 0 g |
| Lima, 2008 | case-control study | 2002-2003 | quantitative FFQ | milk (any type) | age group, origin, oral contraceptives, age at menopause, BMI, energy | Total dairy products: ≤9.1 portions/week |
| Lê, 1986 | case-control study | 1976-1980 | questionnaire | skimmed milk, half-skimmed milk, full-cream, cream, yogurt, cheese, cottage cheese, butter | height, weight, overweight index, total fat intake | Yogurt: 0 g  Cheese: 0 g  Butter: less than daily  Whole milk: 0 g  Skimmed milk: 0 g |
| Shannon, 2003 | case-control study | 1988-1990 | FFQ | skim milk, low–fat cottage cheese,  plain yogurt, frozen yogurt, whole milk, mixed dish with cheese, other cheese, ice cream | age, total energy intake, number of pregnancies, highest level of education | Total dairy products: 0–1.34 servings/day  High fat dairy products: 0 – 0.33 servings/day  Low fat dairy products: 0 – 0.18 servings/day |
| Kato, 1992 | case-control study | 1990-1991 | questionnaire | milk, yogurt, cheese, cream | family history of breast cancer, marital status (single), weight, BMI, age at first birth, the number of births, ages at menarche and menopause, consumption of meats, fish, eggs, oily foods, coffee, black tea, alcohol | Total dairy products: <1-2 servings/week |
| Mobarakeh, 2014 | case-control study | 2009 | questionnaire | high fat milk, high fat yogurt, high fat cheese | age, weight, BMI, waist circumference, educational status, parity, lactation, marital status, menopause, history of estrogen therapy, family history of breast disease or cancera | High fat milk: 0 g  High fat yogurt: 0 g  High fat cheese: 0 g |
| Potischman, 1998 | case-control study | 1990-1992 | in-depth interviews | cheese, ice-cream, peanut butter, butter, whole milk, margarine | age, site, race, education level, combination variable for age at first full-term birth and number of full-term births, oral contraceptive use, average lifetime exercise, exercise at ages 12–13 years, current alcohol consumption | Total dairy products:  >31 times/month  >30 servings/month |
| McCann, 2017 | case-control study | 2003-2014 | FFQ | milk, whole milk, low-fat milk, yogurt, low-fat cheese, other cheese, sweet dairy, cream | age, race, BMI, menopausal status, energy intake, type of milk usually consumed, cigarette smoking  status, family history of breast cancer | Yogurt: 0g  Cream/cheese: 0g |
| Zhang, 2011 | case-control study | 2007-2008 | FFQ | whole milk,  skim/low-fat milk, whole milk powder, skim/low-fat milk powder, yogurt, milk tea, cheese, ice cream | age at menarche, live births and age at fifirst live birth, months of breast feeding, BMI, history of benign breast disease, mother/sister/daughter with breast cancer, physical activity, passive smoking, total energy intake | Total dairy products: 2.98 g/day  High fat dairy products: 0.0 g/day  Low fat dairy products: 0.79 g/day |
| van't Veer, 1989 | case-control study | 1985-1987 | questionnaire | gouda cheese, milk, butter milk, curds, kefir | age, fat and alcohol intake, history of benign breast disease, first- and second-degree familial history, number of cigarettes smoked daily, woman's educational level, ever use of oral contraceptives, age at menarche, age at first full-term pregnancy, parity, BMI, geographic area | Fermented dairy products: 0g  Cheese: 0g  Milk: 0g |
| Ronco, 2002 | case-control study | 1999-2001 | FFQ | Whole milk, skim milk, chocolate milk, ricotta cheese, mozzarella cheese, dambo cheese, parmesan cheese, gruyere cheese, whole yoghurt,  skim yoghurt, butter, chantilly cream, ice-cream | age, years of urban status, education, age at menarche, family history of BC, number of live childbirths, BMI, menopausal status, total energy, total fruit intake | Total dairy products: 949 servings/year  Yogurt: 0 servings/year  Cheese: 260 servings/year  Cream: 0 servings/year  Milk: 0 servings/year  Butter: 0 servings/year  Whole milk: 0 servings/year  Full fat yogurt: 0 servings/year  Skimmed milk: 0 servings/year  Skim yogurt: 0 servings/year |
| Ahmadnia, 2016 | case-control study | 2014-2015 | FFQ | milk (any type) | age, energy, menopausal status, estrogen receptor, progesterone, smoking status | Total dairy products: 0 g/week |

FFQ: food frequency questionnaire; BMI: body mass index; HRT: hormone replacement therapy
