# Supplementary material for: The relationship between dairy products intake and breast cancer incidence: a meta-analysis of observational studies
Source: BMC Cancer. 2021 Oct 15;21:1109. doi: 10.1186/s12885-021-08854-w (PMC8520314; doi:10.1186/s12885-021-08854-w)
Supplement: Supplementary file 2 — Additional file 2 Supplementary Table 2 Quality assessment of studies included. [file 12885_2021_8854_MOESM2_ESM.docx]

**Supplementary Table 2.** Quality assessment of studies included.

| Author, year,  Study (Observational) | **Selection (Out of 4)** | | | | **Comparability**  **(Out of 2)** | **Outcomes (Out of 3)** | | | **Total**  **(Out of 9)** |
| --- | --- | --- | --- | --- | --- | --- | --- | --- | --- |
|  | Representativeness of exposed cohort | Selection of nonexposed cohort | Ascertainment  of exposure | Outcome not present at the start of the study |  | Assessment of outcomes | Length of follow-up | Adequacy of follow-up |  |
| Kaluza, 2020 | 1 | 1 | 1 | 1 | 2 | 1 | 1 | 1 | 9 |
| Farvid, 2018 | 1 | 1 | 1 | 1 | 2 | 1 | 1 | 1 | 9 |
| Fraser, 2020 | 1 | 1 | 1 | 1 | 2 | 1 | 1 | 1 | 9 |
| Genkinger, 2013 | 1 | 1 | 1 | 1 | 2 | 1 | 1 | 1 | 9 |
| Kesse-Guyot, 2007 | 1 | 1 | 1 | 1 | 2 | 1 | 1 | 1 | 9 |
| Knekt, 1996 | 1 | 1 | 1 | 1 | 2 | 0 | 1 | 1 | 8 |
| Shin, 2002 | 1 | 1 | 0 | 1 | 2 | 1 | 1 | 1 | 8 |
| Pala, 2009 | 1 | 1 | 1 | 1 | 2 | 0 | 1 | 1 | 8 |
| Marcondes, 2019 | 1 | 1 | 1 | 1 | 2 | 1 | 1 | 1 | 9 |
| Shin, 2019 | 1 | 1 | 1 | 1 | 2 | 1 | 0 | 1 | 8 |
| Hjartåker, 2010 | 1 | 1 | 1 | 1 | 2 | 1 | 1 | 0 | 8 |
| McCullough, 2005 | 1 | 1 | 0 | 1 | 2 | 1 | 1 | 0 | 7 |
| Gaard, 1995 | 1 | 1 | 1 | 1 | 2 | 1 | 1 | 1 | 9 |
| Wirfält, 2011 | 1 | 1 | 1 | 0 | 2 | 1 | 1 | 1 | 8 |
| Yu, 2019 | 1 | 1 | 1 | 1 | 2 | 1 | 1 | 1 | 9 |
| Franceschi, 1995 | 0 | 1 | 1 | 1 | 2 | 1 | 0 | 1 | 7 |
| Galván-Salazar, 2015 | 1 | 1 | 1 | 1 | 1 | 1 | 1 | 1 | 8 |
| Potischman, 2002 | 1 | 1 | 0 | 1 | 2 | 1 | 1 | 0 | 7 |
| Jayalekshmi, 2009 | 1 | 1 | 1 | 1 | 1 | 1 | 1 | 1 | 8 |
| Hirose, 2003 | 1 | 1 | 0 | 1 | 2 | 1 | 1 | 1 | 8 |
| Bao, 2012 | 1 | 1 | 1 | 1 | 2 | 1 | 1 | 0 | 8 |
| Van 't Veer, 1991 | 1 | 1 | 1 | 1 | 2 | 1 | 1 | 1 | 9 |
| Bahadoran, 2013 | 0 | 1 | 1 | 1 | 2 | 1 | 1 | 0 | 7 |
| Toniolo, 1994 | 1 | 0 | 1 | 1 | 2 | 1 | 1 | 1 | 8 |
| Plagens-Rotman, 2017 | 1 | 1 | 1 | 1 | 2 | 1 | 1 | 1 | 9 |
| Lima, 2008 | 1 | 1 | 1 | 1 | 1 | 1 | 1 | 1 | 8 |
| Lê, 1986 | 0 | 1 | 0 | 1 | 2 | 0 | 1 | 1 | 6 |
| Shannon, 2003 | 1 | 1 | 0 | 1 | 2 | 1 | 1 | 1 | 8 |
| Kato, 1992 | 1 | 1 | 1 | 1 | 2 | 1 | 1 | 0 | 8 |
| Mobarakeh, 2014 | 1 | 1 | 1 | 1 | 2 | 1 | 1 | 1 | 9 |
| Potischman, 1998 | 1 | 0 | 0 | 1 | 2 | 1 | 0 | 1 | 6 |
| McCann, 2017 | 1 | 1 | 1 | 1 | 1 | 1 | 1 | 1 | 8 |
| Zhang, 2011 | 1 | 1 | 1 | 1 | 2 | 1 | 1 | 1 | 9 |
| van't Veer, 1989 | 1 | 1 | 0 | 1 | 2 | 1 | 1 | 0 | 7 |
| Ronco, 2002 | 1 | 1 | 1 | 1 | 2 | 1 | 1 | 1 | 9 |
| Ahmadnia, 2016 | 0 | 1 | 1 | 1 | 2 | 1 | 1 | 1 | 8 |

The observational studies were assessed by the Newcastle-Ottawa Quality Assessment Scale (NOS) checklist.
